# Younger, not better: No influence of age on lung cancer stage at diagnosis in South Africa

**DOI:** 10.7196/AJTCCM.2020.v26i2.075

**Published:** 2020-06-15

**Authors:** Greg Calligaro

**Affiliations:** Division of Pulmonology, Department of Medicine, Groote Schuur Hospital and University of Cape Town Lung Institute, Cape Town, South Africa

**Keywords:** lung cancer, staging, NSCLC

## Editorial

Lung cancer is the leading cause of cancer mortality worldwide, and has its peak incidence in the 7th and 8th decade of life. However, about 2 - 5% of all lung cancer cases occur in younger patients.^[1–3]^ While what constitutes a ‘younger’ patient with lung cancer is not clearly defined in the literature, most studies define it as patients below 40 to 50 years in age. This subgroup of patients is currently a hot area of research interest for many reasons.

Firstly, from the human perspective, lung cancer among younger patients affects breadwinners and parents – in short, individuals in their socioeconomic prime. Their illness has major consequences for families, the wider community and the economy. Secondly, the biology of lung cancer in these patients may be different compared with their older counterparts. Younger patients generally have a much lower cumulative temporal exposure to the traditional carcinogens known to be associated with lung cancer, such as smoking and pollution. Molecular studies show that younger patients with lung cancer are more likely to have tumour genomic activating mutations in epidermal growth factor receptor (EGFR), anaplastic lymphoma kinase (ALK) and human epidermal growth factor receptor 2 (HER2) genes.^[4–6]^ The potential for the use of driver gene specific drug therapy in this group of patients has important implications for prognosis and survival. It has also been suggested that the histological subtypes of lung cancer may be differently represented among younger v. older age groups. And lastly, there is a perception that cancer diagnosis in younger patients may be delayed because of a strong bias towards the consideration of other aetiologies (usually of an infective nature). The high prevalence of late-stage disease at presentation among younger patients has been reported in several studies, which have shown almost half of younger patients have metastatic disease at diagnosis.^[7–9]^ A recent study^[10]^ from Europe reported that 82% of younger patients had stage IIB/IV disease at diagnosis. These findings have obvious implications for survival; we can imagine that such proportions might be even higher in lowresource countries where infections such as tuberculosis (TB) and HIV are prevalent, where empirical treatment for TB is common, and where poor access to radiological and histological investigations is likely to be the norm.

In this issue of the *AJTCCM*, Mhlana and Koegelenberg^[11]^ from the Division of Pulmonology at the University of Stellenbosch and Tygerberg Hospital report a retrospective comparison between patients younger and older than 45 years with lung cancer over a five-year period (June 2012 to December 2017). Of the total cohort of lung cancer patients, 4.8% were in the younger age group. Adenocarcinoma was the predominant histological subtype among non-small-cell lung cancer (NSCLC) in both groups, and there was no difference in the proportion of patients in either group presenting with advanced disease (stage IIIB to IV). Differences in gender, smoking status and mutational analysis (if available) were not presented.

Regardless of age group, these data make for sobering reading: overall, 93% of patients referred to the hospital with lung cancer already had advanced disease. In the 48 younger patients with NSCLC, only a single patient presented with early-state disease, and was treated with curative intent. As targeted therapy for NSCLC is unavailable in the public sector, further interrogation of the biology of these tumours is largely irrelevant. Sadly, this problem is not improving over time: a study from the same centre between 2008 and 2009 reported advanced disease in 89% of patients with NSCLC,^[12]^ while an older report from 1987 at Groote Schuur Hospital in Cape Town also reported that 89% of patients with NSCLC were inoperable.^[13]^ These findings emphasise the cumulative consequences of poor access to healthcare, incorrect diagnosis and delayed referral on lung cancer outcomes in South African patients, which as this study by Mhlana and Koegelenberg^[11]^ shows, affect both younger and older patients indiscriminately.
